# Pathophysiology of Lung Disease and Wound Repair in Cystic Fibrosis

**DOI:** 10.3390/pathophysiology28010011

**Published:** 2021-03-10

**Authors:** Massimo Conese, Sante Di Gioia

**Affiliations:** Laboratory of Experimental and Regenerative Medicine, Department of Medical and Surgical Sciences, University of Foggia, 71122 Foggia, Italy; sante.digioia@unifg.it

**Keywords:** cystic fibrosis, CFTR, airway epithelium, wound healing, EGF/EGFR, epithelial-mesenchymal transition, curcumin, CFTR modulators, mesenchymal stem cells

## Abstract

Cystic fibrosis (CF) is an autosomal recessive, life-threatening condition affecting many organs and tissues, the lung disease being the chief cause of morbidity and mortality. Mutations affecting the *CF Transmembrane Conductance Regulator* (*CFTR*) gene determine the expression of a dysfunctional protein that, in turn, triggers a pathophysiological cascade, leading to airway epithelium injury and remodeling. In vitro and in vivo studies point to a dysregulated regeneration and wound repair in CF airways, to be traced back to epithelial CFTR lack/dysfunction. Subsequent altered ion/fluid fluxes and/or signaling result in reduced cell migration and proliferation. Furthermore, the epithelial-mesenchymal transition appears to be partially triggered in CF, contributing to wound closure alteration. Finally, we pose our attention to diverse approaches to tackle this defect, discussing the therapeutic role of protease inhibitors, CFTR modulators and mesenchymal stem cells. Although the pathophysiology of wound repair in CF has been disclosed in some mechanisms, further studies are warranted to understand the cellular and molecular events in more details and to better address therapeutic interventions.

## 1. Introduction

Cystic fibrosis (CF) is the most common autosomal recessive disorder in the Caucasian population, affecting more than 80,000 people around the world. CF affects many mucosal organs lined by an epithelium, including the lung and gastrointestinal tract, as well as many glands with secretory/adsorptive functions. The lung disease is, however, the major determinant of morbidity and mortality of CF individuals [1]. More than 2000 variants of the CFTR gene have been described, with a genotype-phenotype correlation for only 322 mutations (https://www.cftr2.org/, accessed on 4 March 2021) organized into six classes depending on the fate and function of the CFTR protein [2] (Table 1). According to a newer classification [3], which includes a previous proposal of De Boeck and Amaral [4], CFTR mutations are still categorized into six classes, with the only exception that class I now comprises those of class 1A (no mRNA transcription) and class 1B (stop-codon mutations), both having the same outcome, i.e., absence of the CFTR protein (in case of class IB due to degradation of truncated mRNA by nonsense-mediated decay). Class II (to which the most common mutation—*F508del*—belongs) include mutants that do not overpass the endoplasmic reticulum quality control and are degraded in the proteasome. Class III and class IV mutations are those impairing gating or alter conductance of chloride and bicarbonate ions, respectively, of a CFTR channel correctly transported to the apical membrane. Class V mutations lead to a reduction in the CFTR protein levels due to alternative splicing. Class VI comprises those mutations that destabilize the CFTR protein at the apical membrane. Classes I, II, and III are associated with a more severe phenotype, whereas classes IV, V, and VI with milder phenotypes.

Since the discovery of the *CFTR* gene in 1989, many studies have elucidated the pathophysiological cascade occurring in the bronchi/bronchioli. The CFTR protein is a transmembrane glycoprotein which is comprised of two specular halves (each composed of six membrane-spanning domains and a nucleotide binding domain) connected by a regulatory domain, which makes it unique among the other members of the superfamily of ATP-binding cassette proteins. Functions classically ascribed to CFTR are linked to its activity as a channel of chloride and bicarbonate ions, involved in the proper hydration of airway surface liquid (ASL) [5]. Low or null CFTR protein expressed at the plasma membrane or its dysfunction [4] results in airway surface dehydration and ASL volume depletion, whose pathogenesis is attributable to abnormal ion and fluid homeostasis at the apical side of airway epithelial cells [6]. CFTR is involved also in sodium ions concentration in the ASL by a tonic inhibitory effect on the epithelial sodium channel (ENaC) [7,8], thereby in CF ENaC is hyper-activated and contributes to the liquid hypersorption from the airways [9]. Moreover, loss or dysfunctional CFTR is responsible for defective bicarbonate secretion, resulting in a reduced antimicrobial activity of ASL [10], as well as alterations in rheological properties of mucus which becomes dense with increased viscosity [11]. Altogether, these early events ultimately lead to reduced mucociliary clearance (MCC), mucus accumulation, airway plugging, bacterial colonisation, neutrophil inflammation, progressive tissue damage and decline in lung function in CF airways. A wealth of inflammatory and remodeling mediators has been found at altered levels in CF airways, i.e., in samples of sputum, bronchoalveolar lavage (BAL) fluid and exhaled breath condensate, as well as in the blood [12]. These mediators reflect the recruitment and activation of neutrophils (for example, HMGB-1, IL-1β, TNF-α, IL-8, GM-CSF, IL-17, elastase, myeloperoxidase), activation of adaptive immunity arms (IFN-γ, IL-17, IL-33), and events linked to angiogenesis and fibrosis (VEGF, TGF-β).

Besides its role as a chloride/bicarbonate channel, CFTR is known to be involved in many cellular and tissue processes, such as fetal development [13], epithelial differentiation/polarization [14], regeneration [15], tight junction (TJ) formation [16] and epithelial–mesenchymal transition (EMT) [17]. The presence of mutated CFTR is associated with the dysregulation of differentiation and repair, eventually leading to cancer [18], that was described as “a wound that does not heal” [19]. Other alterations associated with CFTR loss/dysfunction are implicated in the maintenance of apical-basal polarity of the airway epithelium and cytoskeletal organization. The barrier function of the airway epithelium is guaranteed by integrity by the apical junction complexes (AJC), consisting of occluding TJs, anchoring adherens junctions (AJs), desmosomes, and gap junctions (GJs). It is well described that mislocalization of TJ proteins, disorganized actin cytoskeleton and lack of actin stress fibers are present in CF cells [20,21,22,23]. The link to these alterations was found to be the interaction of CFTR with a molecular complex, tethering CFTR to the apical side of airway epithelial cells. The scaffolding protein Na^+^/H^+^ exchanger regulatory factor isoform 1 (NHERF1) is essential to maintain CFTR at the apical location through its interaction with ezrin, an A-kinase anchoring protein, that tethers PKA in the proximity of CFTR, allowing cAMP-dependent control of chloride efflux [24,25,26,27,28,29]. On the other hand, a low transepithelial resistance (TEER), a measure of epithelial tightness, indicative of TJ disorganization, has been observed in CF bronchial epithelial cells as compared with a wt-CFTR expressing cells [14,30,31]. Castellani et al. [16] confirmed these previous results on TJ disorganization in CF cells by the lack of TJ proteins at cell-cell contacts (occludin, ZO-1, claudin 1, and JAM-1), and showed also that NHERF1 or CFTR overexpression in CFBE41o- cell (*F508del* homozygous) monolayers induced the reorganization of TJ proteins at the level of intercellular junctions and reduced the paracellular permeability to small solutes. Interestingly, they also found that in CFBE cells, ZO-1 and occludin were localized to the nuclei, whereas the plasma membrane signal was negligible [16], indicating that CFBE cells present a dislocation of TJ proteins at the nuclear level in basal condition. In keeping with these data, Ruan and colleagues [32] have shown that in a three-dimensional (3D) epithelial cell culture model, CFTR interacts with ZO-1 at TJ levels and keeps constrained the transcription factor ZO-1 nucleic acid binding protein (ZONAB) at the same location. Upon CFTR inhibition or knockdown, ZO-1 expression is reduced and the translocation of the transcription factor ZONAB from TJs to the nucleus is induced, and an increased proliferative activity occurs.

GJs are formed by connexins (Cxs), which assemble in the plasma membrane to form hemichannels or connexons, that dock to similar connexons on the neighboring cell allowing GJ intercellular communication (GJIC). By allowing the passage of ions and small solutes, GJs are involved in the regulation of proliferation/differentiation and the maintenance of tissue homeostasis [33,34] as well as in CFTR-related epithelial functions [35,36]. CFTR has been described to interact with Cxs to regulate their trafficking and regulation of GJIC by pro-inflammatory mediators [37]. To note, it was observed that Cx43 showed perinuclear localization in CuFi-5 cells (*F508del* homozygous), while Cx26 localization was unaffected, and both Cxs were at the correct position in non-CF NuLi cells [31], further demonstrating a defect in cell-to-cell contacts in CF airway epithelia.

All these observations link CFTR to epithelial tightness and thus to the modulation of epithelial differentiation and proliferation, as we shall see in the following Sections dedicated to epithelial wound repair and EMT.

### Events Involved in Epithelial Repair and EMT

**Figure 1:**
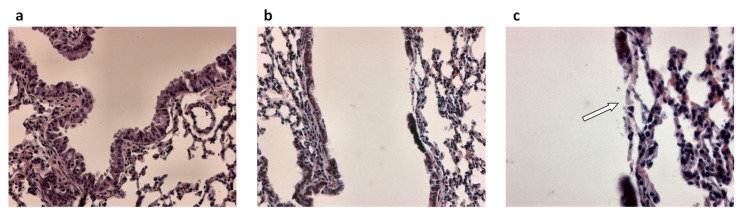
Airway epithelial damage in the airways following infection with *P. aeruginosa*. (**a**) Hematoxylin and eosin-stained lung section from control mice and (**b**,**c**) hematoxylin and eosin-stained lung sections from *P. aeruginosa* infected mice 48 h post-intratracheal instillation at a dose of 1 × 10^5^ colony forming units. Panel **c** is an enlargement of panel **b**. Original magnification ×20 (**a**,**b**) and ×40 (**c**). White arrow in (**c**) indicates loss of the airway epithelium.

In principle, epithelial repair follows different types of injury. In the case of CF, the damage to the airway epithelium can be caused by respiratory infections, in particular *Pseudomonas aeruginosa* (Figure 1) [38] and inflammatory mediators [39]. Also bacterial products secreted by *P. aeruginosa*, triggered by the quorum sensing (QS) network and involved in bacterial virulence, pathogenicity, and biofilm formation, have been shown to reduce airway epithelial repair rates [40,41,42]. On the other hand, heightened production of inflammatory mediators and reductions in anti-inflammatory molecule secretion by inflammatory and epithelial cells in CF lungs play a role in injury and remodeling of respiratory epithelia [43,44,45].

In the airways, epithelial repair follows general rules of reconstitution whose hallmarks are [46]: spreading and migration of neighboring epithelial cells using filopodial and lamellipodial extensions, a further step characterized by migration and proliferation of progenitor cells and, finally, differentiation. Recently, EMT induction, during which epithelial cells transform into mesenchymal-like cells, has been recognized as essential in physiologic and pathologic repair [47,48]. During EMT, epithelial cells lose their epithelial markers (i.e., E-cadherin) and present a migratory phenotype to re-epithelialize the wound. Fibroblast growth factor (FGF), epidermal growth factor (EGF), hepatocyte growth factor (HGF), and transforming growth factor (TGF)-β are growth factors orchestrating wound repair steps and are also involved in the initiation and regulation of the EMT [47].

The epithelium maintains its integrity through cell surface protein complexes, which contribute to form epithelial cell–cell junctions. The reorganization of the cytoskeletal architecture and polarity complexes, which result in cell shape changes, cell elongation, membrane protrusions and front–rear polarity, are essential in EMT and enable directional migration. Upon the initiation of EMT, junctions composed in the AJC are deconstructed and the junction proteins are relocalized and/or degraded [49]. A decreased claudin and occludin expression, and the diffusion of ZO-1 from cell–cell contacts has been described in early stages of EMT [50]. The findings of the nuclear localization of occludin and ZO-1 in CFBE cells [16] can be now viewed in the context of partial EMT state of CF airway epithelial cells (see below). E-cadherin—one of the main components of AJs—once cleaved and detached from the plasma membrane can be degraded [51]. Therefore, β-catenin is released from E-cadherin and act as transcription factor in the presence of WNT signaling which protects it from degradation [52]. The decrease in E-cadherin levels can even cause the accumulation of p120 catenin (also known as catenin δ1) in the nucleus where it works as transcription factor [53]. EMT initiation is also associated with disruption of desmosomes [50,51], and the decrease of Cx levels which in turn induce the loss of GJs [54]. During EMT progression, a decrease in the junction proteins expression at transcriptional level is observed, and this further stabilizes the loss of epithelial junctions [55,56]. Moreover, the apical–basal polarity, which is mediated by molecular complexes physically and functionally integrated with the cell junction architecture, is lost.

Another key role of EMT programme is to provide cells with the ability to migrate by invading through ECM. This process requires the reorganization of cytoskeleton so as to permit the dynamic elongation and directional motility [51,57,58]. Cells produce several types of membrane protrusion, which are rich of actin and facilitate cell movement and act as sensory extensions of the cytoskeleton. These projections include lamellipodia, which are sheet-like membrane protrusions, and spike-like extensions called filopodia at the edge of them [59]. Invadopodia degrade ECM by proteolytic activities, thus facilitating cell invasion [59,60]. The family of RHO GTPases is involved in these processes with RHOA promoting actin stress fibre formation and RAC1 and CDC42 promoting the formation of filopodia and lamellipodia. The conversion from apical-basal polarity (epithelial cells) to front-rear polarity (mesenchymal cells), which is one of the main aspects of EMT, involves RHO GTPases [61,62].

The repression of genes which are fundamental for the epithelial structure (e.g., E-cadherin) is counterbalanced by the activation of genes encoding proteins involved in mesenchymal adhesion such as N-cadherin (mesenchymal neural cadherin). Depending on the cell type and the extent to which cells advance through an EMT programme, cells undergoing an EMT may begin to express vimentin, to suppress cytokeratin, to shift expression of key integrins and so forth. These changes in the intermediate filament composition enable cell motility as vimentin can interact with motor proteins [63].

Remodelling of the ECM and changes to cell interactions with the ECM are essential in the initiation and progression of EMT. While acquiring a mesenchymal phenotype, epithelial cells lose their interaction with basal membrane and communicate with an inflammatory ECM.

As integrin complexes enable cells to receive ECM signals and integrate those elicited by growth factors, some epithelial integrins are down-regulated while others are activated in EMT. Some of these newly expressed integrins have key roles in EMT progression [51]. Thus, α_6_β_4_, that mediates contacts with the basement membrane, is epigenetically down-regulated [64], whereas β_1_ integrins increase during EMT [65,66,67,68]. During wound repair, β1-integrin subunit was found to be expressed by repairing cells on their basolateral side as well as on the apical side [69]. Kim and colleagues [65] demonstrated the crucial role of the laminin receptor α_3_β_1_ integrin in E-cadherin turnover and the remarkable crosstalk and interdependence of TGF-β signalling and β-catenin–SMAD signalling systems, in the control of EMT. Also the fibronectin receptor α_5_β_1_ integrin expression is augmented during EMT increasing cell adhesion to fibronectin, the expression of which is also activated during EMT, and promoting cell migration and protection from apoptosis. The increased expression of the β_1_ integrins and their ligation of collagen type I initiates a signaling cascade, leading to disassembly of the E-cadherin adhesion complex and the nuclear translocation of β-catenin, ultimately determining cell proliferation [68]. A strict connection of changes to the integrin repertoire and ligation as well as intracellular signals and increased expression of metalloproteinases (MMP2 and MMP9) has been demonstrated [49,70,71]. MMPs will enhance ECM protein degradation where integrin adhesion receptors focally interact with inflammatory ECM proteins, thus enabling invasion [51,59,72]. Other effects of MMPs related to the EMT activation are related to the trimmering of the extracellular domain of E-cadherin, thus contributing to the loss of AJs [70], and to increased SNAIL1 expression operated by increased levels of reactive oxygen species [73]. Finally, it has to be recognized that some growth factors are stored in the ECM and that localized ECM degradation may release them, such as is the case for TGF-β that is present in a latent form and is activated by MMPs and α_v_β_6_ [74,75]. TGF-β, in turn, stimulates the expression of collagens and fibronectin, which are involved in the matrix remodeling.

EMT is induced by an interplay of soluble growth factors such as HGF, members of the TGF (e.g., TGF-β) and FGF families, insulin-like growth factor (IGFs, e.g., IGF-1), EGF as well as extracellular matrix such as collagen or hypoxic conditions. These factors activate signaling pathways leading to either expression or post-transcriptional and post-translational modification of EMT-associated transcription factors (EMT-TFs) [76,77]. Three main families of EMT-TFs have been described with the SNAI (SNAI1/Snail and SNAI2/Slug), ZEB (ZEB1 and ZEB2), and TWIST (TWIST1 and TWIST2) nuclear proteins, playing pivotal roles in the orchestration of EMT [78]. These EMT-TFs have been shown to interact with a variety of proteins involved in transcriptional regulation including proteins that function in epigenetic modification, forming together regulatory complexes.

In the airways, the final step of wound repair is the terminal differentiation which means the reconstitution of a pseudostratified epithelium with mucus-secreting cells (goblet), secretory (Club cells), ciliated cells, PNEC (pulmonary neuroendocrine cells), and basal cells. Recent studies have identified another cell type which has been called “ionocyte” and expresses higher levels of CFTR than other airway cells do [33,34]. Ionocytes derive directly from basal cells (BCs), some of which appear to function as classic multipotent stem cells, while other BCs are thought to be progenitors already committed to a ciliated or secretory fate [79,80]. As genetic (single-cell RNA sequencing) and more detailed histological and functional studies advance, not only ionocytes but also other rare cell types derived from BCs (PNEC, tuft cells, mucous ciliated cells and deuterosomal cells) are being studied during airway epithelium regeneration and repair [81,82].

**Figure 2:**
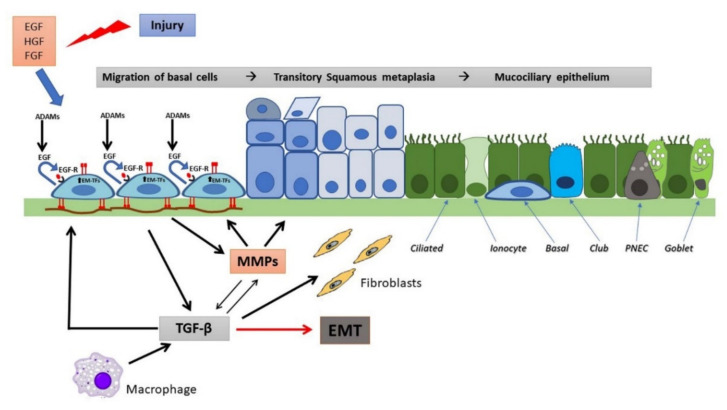
Wound repair and regeneration in a non-CF airway epithelium. Following injury, many cytokines and growth factors (e.g., EGF, HGF, FGF) are secreted in the wound repair microenvironment, which incite in basal cells a migratory and proliferative phenotype. Shedding of EGFR ligands (e.g., EGF) by ADAMs and binding to EGFR in an autocrine or iuxtacrine manner are key events involved in stimulation of cell migration and proliferation. Afterwards, MMP secretion by regenerating epithelial cells and subsequent TGF-β activation lead to genetic expression changes, related to EMT stimulation and activation of EMT-transcription factors (EMT-TFs). Epithelial cells and macrophages release TGF-β that induce ECM component deposition by epithelial cells and stimulate fibroblast activation, resulting in further matrix deposition. These events provoke alterations in junctional complexes and reorganization of actin cytoskeleton (not shown), modification of various integrin expression with β_1_-integrins increase at basal side and ectopic expression on the apical side, deposition of inflammatory ECM glycoproteins (e.g., fibronectin is shown) and its remodeling exerted by MMPs. TGF-β and MMPs enhance each other in a positive way. This process proceeds with the formation of a squamous stratified epithelium and subsequent pseudostratification and mucociliary differentiation.

The processes of epithelial repair are modulated by several growth factors, including EGF and related cytokines (TGF-α, amphiregulin, heparin-binding EGF), which through EGF receptors (EGFR), produce motility, proliferative responses, differentiation and survival [43,83,84]. MMPs and a disintegrin and metalloproteases (ADAMs) release from the cell surface EGFR ligands, including TGF-α, heparin-binding EGF (HB-EGF) and amphiregulin (AREG), that in turn can bind and activate EGFR in an autocrine or paracrine manner, while the transmembrane (pro) form may activate EGFR in adjacent cells (juxtacrine) [85]. During migration, cells attach to a provisional matrix of fibronectin and other extracellular matrix (ECM) components such as fibrin and fibrinogen. Platelet-derived growth factor (PDGF) and TGF-β act as the main modulators of ECM deposition by attracting and activating fibroblasts, in this way regulating airway repair [86,87]. Migrating epithelial cells overexpress MMPs to degrade the provisional matrix which provides novel attachment sites for lamellipodia. MMP-9 (gelatinase B), MMP-3 and MMP-11 (stromelysin 1 and 3, respectively), and MMP-7 (matrilysin), have been implicated in the matrix remodeling and acquisition of a typical epithelial-mesenchymal phenotype [88]. It has been suggested that the high expression and activation levels of MMPs can be regulated by an increase in IL-8, a pro-inflammatory cytokine [89]. TGF-β plays also a role in this remodelling process by up-regulating MMP-2 [90]. Furthermore, epithelial repair is stimulated by other growth factors, namely insulin, IGFs, HGF, keratinocyte growth factor (KGF), calcitonin gene-related peptides, and the cathelicidin LL-37 peptide. HGF and KGF acts as chemotactic and growth-stimulating factors. They also stimulate the synthesis of ECM and facilitate interactions with MMPs through specific cell receptors [91,92]. The main features of regeneration and wound repair in a non-CF airway epithelium are represented in Figure 2.

In this review, we focus our attention on pathological processes occurring in the CF airways, with a particular focus on regeneration and wound repair.

## 2. Pathological Processes in the CF Airways

Dorothy Andersen was the first to describe the condition named “cystic fibrosis of the pancreas” and observe in 49 pediatric cases at the levels of the lungs some pathological features characteristics of CF: mild tubular dilatation of small bronchi and bronchioles, the presence of mucopurulent material in the larger bronchi and trachea with “some congestion of the underlying mucosa”, and multiple small abscesses in the smaller bronchi [93]. Her report also described the infection of airways and she observed that most of the patients in her study died of pneumonia before the age of 6 months. She and others also described squamous metaplasia of the respiratory epithelium, to be related to vitamin A deficiency and a factor in perpetuating the respiratory infections due to the absence of the protective role of mucus [94,95].

Pathological changes occurring in the airways of toddlers and older children with CF are characterized by mucopurulent plugging of small and medium size bronchioles and development of bronchiectasis, secondary to proteolysis and chondrolysis of airway support tissues [96]. The dilated airways contribute to reduced mucociliary and cough clearance and the persistence of mucus inspissation and endobronchial inflammation.

Bacterial infections and the chronic hyper-inflammatory response generated as a consequence, lead to ensuing repairing mechanisms in response to the epithelial damage [97,98,99]. Neutrophils represent the main inflammatory cell type found into the lumen of CF airways and they have been shown to contribute to the pathophysiology of CF lung disease. Indeed, these immune cells, once entered into the CF airways, produce damaging mediators (proteases, reactive oxygen species, elastase) [100] that induce epithelial apoptosis [101] and/or premature senescence [102], and herald the remodeling of the airway epithelium by upregulating mucin expression and inducing goblet cell metaplasia [103,104]. Besides the hyperactivation of the CF airway epithelium, which triggers neutrophil attraction and activation in the CF airways by producing cytokines and chemokines [105], other immune cells and mediators have been found to be involved. Elevated levels of IL-17 and IL-23 in the sputum of CF patients, and particularly in those chronically infected with *P. aeruginosa*, implicate a role for Th17 cells in the persistent neutrophil infiltration in CF lung disease and chronic infection with *P. aeruginosa* [106]. Pretreatment of immortalized CF bronchial cells (NuLi) with IL-17 determined much greater IL-8 secretion in response to an agonist of NOD1, a cytosolic innate immune receptor, and *P. aeruginosa* diffusible material, identifying an amplification mechanism by which CF epithelial cells may trigger the inflammatory response to bacterial ligands [107]. Furthermore, the treatment of primary CF bronchial epithelial cultures with IL-17 increased production of IL-8, IL-6 and granulocyte macrophage colony-stimulating factor, confirming a positive feedback element in CF airway inflammation involving adaptive immunity.

The structural alterations accumulating with time in CF airways include hyperplasia of goblet and basal cells [39,97,108,109], squamous metaplasia [109,110], increase in epithelial height [39,108,111], cell shedding [97,98,108,109,111], and increased thickness of reticular basement membrane (RBM) [97,112]. Airway ECM remodeling and thus structural changes follow enhanced degradation of ECM proteins such as elastin, collagen, and glycosaminoglycans. These alterations are associated with a marked and early protease/antiprotease imbalance and the release of unopposed amounts of neutrophil elastase (NE), MMPs and other proteases that are involved in tissue damage and remodeling [113,114,115,116]. In vivo data show considerable evidence of an imbalance of MMPs and their inhibitors (tissue inhibitors of MMP, TIMPs) in the CF airways with prevalence and activation of MMPs [114,117,118,119]. The action of MMPs, as well as of serine (NE) and cysteine (cathepsins) proteases, secreted by epithelial cells, macrophages and the recruited neutrophils, in the pathogenesis of CF airway disease is complex and multi-faceted, including the enhancement of mucin/mucus production and secretion, the activation of PARs (protease-activated receptors) leading to proinflammatory signaling, the trans-activation of other proteases by cleaving pro-domains and degrading cognate antiproteases, the aggravation of basic CF ion transport defects by the proteolytic degradation of CFTR and activation of ENaC, and the cleavage of various host protein substrates precipitating either activation (in the case of some proinflammatory cytokines) or inactivation (in the case of some antimicrobial peptides and surfactant proteins) [120].

Some other molecules involved directly in the remodeling of CF airways have been identified and studied. TGF-β, which has been found at elevated levels in blood (plasma) and BAL levels in CF patients and have been associated with pulmonary exacerbations [121,122], plays multiple roles in the pathogenesis of CF lung disease [123]. Besides downregulation of chloride transport in airway epithelial cells by acting negatively on CFTR and on Calcium-activated Chloride Conductance (operated by TMEM16A) [124,125,126], TGF-β signaling may drive goblet cell hyperplasia and increased mucin secretion, as indicated by studies in mouse models [127,128]. Immune and nonimmune cells, including airway epithelial cells [129,130], can secrete the latent form of TGF-β, which, upon activation orchestrates the lung immunity [131,132]. The increased RBM thickness in children with CF was found to be significantly related to BAL concentrations of TGF-β1 but unrelated to the raised levels of inflammatory cells and other cytokines [113]. Importantly, lung samples obtained from CF patients showed a significant peribronchiolar remodeling associated with prominent myofibroblast differentiation and fibrosis, i.e., TGF-β-dependent processes [129,133].

The EGFR is involved in mucin expression and secretion in the CF airways [134]. Indirect activation of MMPs and ADAMs by TNF-α stimulation can induce the EGFR pathway by evoking EGFR ligand shedding and inducing the EGFR pathway [135,136,137] in epithelial cells [138]. Both EGFR [139,140] and IL-13 receptor [141] have been associated with mucous cell metaplasia and mucin synthesis. More recently, the EGFR has been functionally coupled to ADAM17 at the level of the airway epithelium, and the EGFR/ADAM17 axis and its signaling pathway has been linked to TGF-α and AREG release and mucin expression [142]. AREG autocrine signaling affects mucus expression [143] and cytokine secretion [144], whereas its paracrine signaling has been linked to TGF-β-induced fibrosis [145]. Moreover, CF bronchial epithelial CFBE41o- cells displayed an enhanced ADAM17-mediated shedding of AREG compared with genetically identical cells with induced wt-CFTR expression and this correlated with enhanced apical presentation and phosphorylation of EGFR [146]. Further studies are necessary to clearly determine the role of EGFR/ADAM17 axis in CF, wound repair and other associated pathological hallmarks of lung disease. Nevertheless, all these changes indicate that epithelial differentiation and likely migration occurring to repair damage are somehow altered in CF airways. In the following Sections, we shall review those studies trying to elucidate the underlying mechanisms of these alterations, with particular reference to regeneration and wound repair.

## 3. Airway Epithelial Regeneration, Wound Repair and CF

The complete regeneration of a wounded airway epithelium is a complex phenomenon, including epithelial wound repair and differentiation to reconstitute a functional epithelium [147]. The regeneration occurs when stem/progenitor cells interact with factors that regulate this process. Indeed, basal cells at the wound edge de-differentiate, flatten, then migrate to cover the denuded area. In the first phases of this process, cell migration seems to predominate over cell proliferation. After this step, epithelial cells in the repairing wound start to proliferate. Although the epithelial integrity is reconstituted by cell migration and proliferation [148,149], the regeneration process is not complete. In a successive step, a transitory squamous metaplasia occurs followed by progressive re-differentiation to restore a pseudostratified and functional mucociliary epithelium [150]. An essential role in successful wound repair is given by interactions between epithelial cells themselves, fibroblasts and the ECM [147,151]. The deposition of a provisional ECM, the cell-EMC relationship through epithelial receptors, and the remodeling of the ECM by proteases contribute not only to epithelial wound repair by modulating cell migration and proliferation, but also to the differentiation of repairing cells. For cells to migrate, the interaction between ECM glycoproteins (fibronectin, vitronectin, laminin and type IV collagen) and their cellular receptors (integrins) is crucial [152], in particular fibronectin and the corresponding receptor, i.e., the α_5_β_1_ integrin, play an important role in the process of airway epithelium wound repair [69,153]. MMPs exert an important role in the remodeling of ECM during cell migration, since during this process cells attach to ECM proteins in the front of advancing edge and release their interaction at the rear [147]. MMP-9, as well as MMP-3 and MMP-11 have been found to be highly expressed by basal migratory cells at the wound edge [154,155,156]. Notably, it was found that MMP-3 was overexpressed and overactivated during the wound repair process, with the maximal production observed at wound closure by CK14^+^ basal cells, which were also expressing the mesenchymal marker vimentin, suggesting that repairing cells acquire a mesenchymal phenotype necessary for cell migration [156]. Others have described that MMP-7 is constitutively produced in airway epithelium, is increased in CF epithelial cells, and its expression was increased in migrating airway epithelial cells in wounded human and mouse trachea [157]. TGF-β is among the factors that are secreted by epithelial cells during the wound repair that have a complex role by inducing ECM protein deposition and modifying integrin and MMP expression. TGF-β was shown to induce both fibronectin mRNA expression by airway epithelial cells and its secretion [158]. On the other hand, TGF-β decreased the sheet migration of bronchial epithelial cells while increasing their attachment to matrix-coated dishes [159]. Mechanical wounding of bronchial epithelial cell monolayers was shown to induce integrin-dependent activation of endogenous TGF-β1 and to slow the degree of wound closure as exerted as cell sheet [160]. In a more complex model, i.e., during differentiation of human nasal epithelial cells, the addition of exogenous TGF-β1 accelerated wound repair via MMP-2 upregulation [90]. Thus, complex interactions among repairing cells, ECM proteins and their receptors as well as secreted factors occur during wound repair and regeneration of the injured airway epithelium, and the elucidation of these processes depends on the in vivo, ex-vivo and in vitro models that are used.

**Figure 3:**
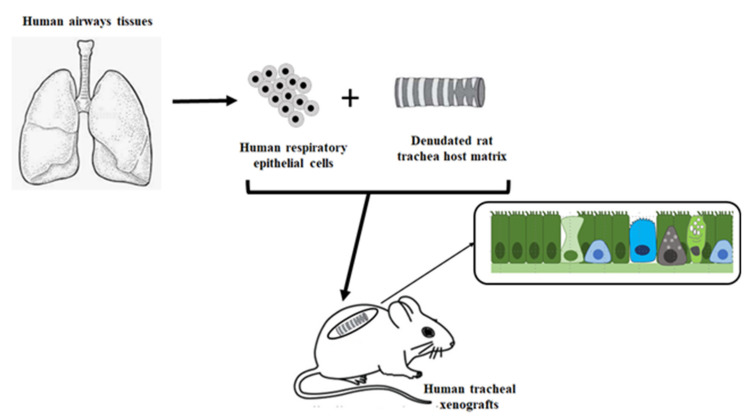
The “air-opened” nude mouse xenograft model. Dissociated airway epithelial cells are seeded into the lumen of a denuded rat trachea tied at their end to sterile tubing. The assembly is subcutaneously implanted in the flank of a recipient nude mouse. Epithelial repair and regeneration steps result in the generation of a well-differentiated mucociliary epithelium (inset).

**Figure 4:**
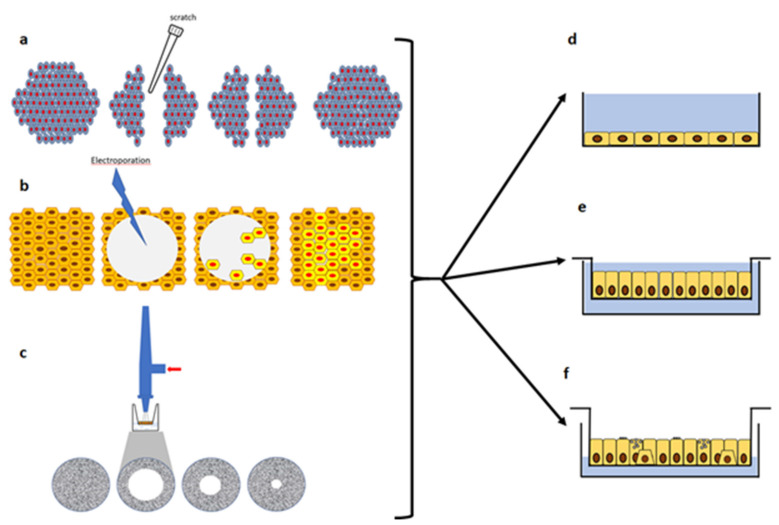
Methods of wound injury in different models of airway epithelial cell cultures. (**a**) Mechanical injury by the pipette tip (scratch assay). (**b**) Circular lesion produced by lethal electroporation. (**c**) Circular wounds obtained by an airbrush linked to a pressure regulator. (**d**) Submerged cultures of immortalized cell lines on plastic. (**e**) Polarized cultures of immortalized and primary cells seeded onto semipermeable filters. (**f**) ALI cultures on semipermeable filters. While immortalized cell lines polarize but not differentiate at ALI (e.g., Calu-3), primary airway epithelial cells differentiate at ALI into a pseudostratified epithelium presenting ciliated, basal, secretory, and mucus-producing goblet cells.

The regeneration and wound repair of the airway epithelium in CF has been studied in models of different complexity and outcomes. Ex-vivo xenograft models have been used in the context of re-differentiation of a native airway epithelium leading to regeneration and mucosa reconstitution by basal-like cells [161]. On the other hand, in-vitro models have been used to study wound repair through simplistic models of injury and repair [138,162,163,164,165,166]. These studies have been mainly conducted in submerged cultures on plastic, with either immortalized or primary cells lines, leaving aside all the fact that in vivo we deal instead with a polarized pseudostratified airway epithelium. Moreover, heterogenous results were obtained when submerged cultures were interrogated as regard inflammation and mucus production [167]. In other studies, polarized cultures were employed as well as air-liquid interface (ALI) cultures. ALI cultures of primary airway epithelial cells (derived from bronchi or nose) represent of what is more proxy to the in-vivo situation. Indeed, in this model, human airway epithelial cells reproduce the 3D structure of well-differentiated, non-proliferating, airway epithelium with basal, secretory and ciliated cells in a context devoid of exogenous infection or inflammation [168]. Using these protocols, primary airway epithelial cells are seeded onto the apical chamber of semipermeable inserts coated with type IV collagen and with media in the basal chamber. Upon reaching cell confluency, the apical medium is removed and the basal medium switched to ALI-specific media [169]. Cells are then cultured for 2–4 weeks to allow proper differentiation and polarization. Polarized monolayers and ALI cultures form AJC, whose organization can be measured by TEER. All these models and outcomes have been presented in Table 2 and Figure 3 and Figure 4.

### 3.1. Airway Epithelial Regeneration in Xenograft Models

Epithelial regeneration and airway mucosa reconstitution was studied in a xenograft model by which epithelial cells obtained from nasal polyps were seeded onto denuded rat tracheas [170], in a way that the reconstitution of a human airway epithelium occurs upon exposure to the external milieu in the same way as in adult human airways [171] (Figure 3). By day 3, a flattened non-ciliated and poorly differentiated leaky epithelium covered the trachea surface. By the end of the first week, a protective squamous stratified epithelium appeared. During the following weeks, cell proliferation which was intense in the previous phases, decreased markedly and the epithelium was now composed of more differentiated cell types, including columnar, secretory and ciliated cells. CFTR expression was detected at the apical domain of ciliated cells only when the epithelium was fully differentiated. These results were subsequently confirmed by studies with 3-D spheroid structures that were shown to be able to repopulate denuded mice tracheas and reconstitute a well-differentiated human airway surface epithelium [172].

The sequential phenotypic changes in integrin subunit expression was studied by Pilewski and colleagues in a xenograft model of bronchial epithelium [153]. After mechanical injury, there was increased expression of the α_v_-, β_5_-, β_6_, and α_5_-integrin subunits on the migrating cells at the edges of surface epithelial wounds, while during the later phase of repair, they found sustained upregulation of the α_v_β_5_ and α_v_β_6_ integrins. Thus, the integrins binding to inflammatory fibrinogen/fibronectin/vitronectin, specifically the α_v_- and α_5_ as well as the β_5_- and β_6_-subunits, are upregulated during wound repair. Interestingly, in CF xenografts the expression of the α_5_-, α_v_-, β_5_, and β_6_-integrin receptor subunits was the same as in areas of non-CF epithelium from bronchial xenografts, However, there was increased expression of the α_v_-, β_5_-, and β_6_-subunits and no significant expression of the α_5_-subunit within the patchy areas of epithelial injury.

To understand better the molecular determinants involved in the normal regeneration process, Coraux et al. [89] showed the role of MMPs and IL-8 during human non-CF airway surface epithelial regeneration. IL-8 mRNA expression was found at high levels during the cell migration (step I), lowered at proliferation step (II), and remained low at step III (epithelial pseudostratification) and step IV (epithelial mucociliary differentiation). The behavior of MMP-7 and MMP-9 mRNAs was the opposite, with MMP-7 and MMP-9 mRNA levels that increased significantly up to 3.9-fold during steps III and IV and 2.9-fold during step IV, respectively. Hajj and colleagues [15] demonstrated that the regeneration of CF airway epithelium followed the same steps (I-IV) as in non-CF human airway epithelium [89,170]. However, the CF epithelium was higher than the non-CF epithelium. Moreover, at the end of the study (day 25), the majority, i.e., 80%, of non-CF grafts were covered by a well-differentiated epithelium, whereas only 18.7% of CF grafts presented this feature. In the second step, CF epithelial cells were more proliferating than non-CF cells and this phenomenon was paralleled by an increase in IL-8, MMP-7, MMP-9 and TIMP-1, implicating these mediators in hyper-proliferation and suggesting that heightened proliferation is involved in the delay observed in CF epithelial differentiation.

In consideration that airway regeneration takes place in the adult xenograft model, some studies attempted to identify and trace progenitor cells of the human airway epithelium. By coupling the technique of retrovirus-mediated gene transfer with a xenograft model of proximal human airway allowed to identify human lung epithelial stem cells that are capable of substantial self-renewal and have pluripotent potential, as testified by the fact that a substantial number of clones showed transgene expression in basal as well as differentiated columnar cells [173]. Using the same approach, Engelhardt and colleagues [174] conducted clonal studies that led to the identification of a pluripotent progenitor cell within the airway epithelium which also retains the capacity for gland development, and determined that more than one progenitor cell is involved in gland formation.

Human embryonic and fetal lung tissue xenografts in immunodeficient mice (SCID) have been instrumental in our understanding of the early steps of inflammation and tissue remodeling in CF airways [161,175]. In this model, proximal airway primordia grew rapidly and differentiated after 6–12 weeks into tracheal structures, including a pseudostratified mucociliary surface epithelium with submucosal gland network [176]. By using the SCID-hu model, host grafts denuded of native epithelium were seeded with either differentiated airway epithelial cells or embryonic lung stem cells, giving rise in both cases to a fully differentiated mucociliary epithelium [177], indicating that this model could be useful as a functional in vivo assay for sorted candidate subsets of such progenitors. To this end, host human fetal xenografts were deprived of their own native epithelium and seeded with either aquaporin-3 (AQP3)^+^ basal cells or AQP3^-^ suprabasal cell subpopulations from well differentiated human fetal airway xenografts [178]. Interestingly, both sorted subpopulations could restore a well-differentiated, pseudostratified mucociliary surface epithelium and functional submucosal glands, although AQP3^−^ cells were endowed with faster engraftment, suggesting their inclusion of more committed progenitors.

### 3.2. In-Vitro Models of Injury and Repair with Submerged Cultures

That an alteration in wound repair was associated with CFTR lack or dysfunction was initially demonstrated by determining a circular lesion produced by lethal electroporation on cell monolayers and evaluated by using electric cell substrate impedance sensing (ECIS) combined with phase-contrast imaging [163] (Figure 4). Normal human bronchial epithelial cells (NHBE) cells and Calu-3 cells (of serous gland origin) were either treated with CFTR-inh172, a CFTR inhibitor, or CFTR expression was silenced by constitutive expression of CFTR-specific short hairpin (sh)RNA, and in both cases a delay in wound closure was reported, indicating that CFTR appears to be involved. These results were due to the slowing of migration rather than to proliferation. The importance of the CFTR role was also confirmed in a CF cell line (UNCCF1T), obtained from a CF patient homozygous for *F508del*, which showed a delayed wound closure as compared to NHBE cells. Finally, the delay in wound closure correlated with a reduction in lamellipodia protrusion during wound closure. Interestingly, UNCCF1T and NHBE cells used in this study presented abundant expression of the basal cell markers CD151 and cytokeratin 5, as well as the proliferative marker EGFR, suggesting that these cell lines represent useful in vitro model systems for investigating the role of CFTR in bronchial epithelial wound repair at early stages. Overall, these initial studies supported the conclusion that the role of CFTR in wound repair was dependent on its transport activity, rather than other functions of the CFTR protein.

A scratch assay has been largely reproduced and consists in mechanical injury of an airway epithelial cell monolayer by a pipette tip (Figure 4). The reconstitution of epithelial integrity, is mainly determined by cell migration and proliferation [138,162,179]. By using this in vitro assay, Trinh and colleagues demonstrated that wound repair and cell migration in CF (CuFi-1) bronchial cell monolayers were slower than in non-CF (NuLi-1) cell monolayers [162]. Further studies showed that the delay in CuFi-1 wound closure was likely a defect in cell migration rather than in cell proliferation [164]. The involvement of CFTR in cell migration has been further confirmed by other studies with endometrial cells [180], keratinocytes and skin wound repair studies [181,182,183]. Interestingly, this delay was confirmed when primary CF and non-CF airway epithelial cells obtained from both bronchi and nose were compared [162]. After verifying that CFTR plays an important role in wound closure, cell migration and proliferation by means of genetic and pharmacologic approaches, the authors showed that a CFTR corrector (VRT-325) was able to enhance the wound repair process in monolayers derived from CFBE-F508del and primary CF bronchial cells. A further work by the same group demonstrated that Orkambi^®^ (Vertex Pharmaceuticals, Boston, MA, USA, CFTR VX-809 corrector + VX-770 potentiator) improved wound repair in primary cultures of CF airway epithelial cells [184], as we shall discuss in the Section 4 “Modulation of wound repair in CF”.

The scratch assay was instrumental in determining whether CFTR levels corresponded to certain wound closure rates [165]. Wound repair was found to be significantly slower in CFBE cells compared to corrected CFBE cells (CFBE41o-pCep4, overexpressing wt-CFTR), but the comparison between CFBE and 16HBE cells showed no significant difference in the repair. Also CFSME cells (CF submucosal gland) showed a significantly reduced wound repair rate in comparison to Calu-3 cells (non-CF submucosal glands). No differences were found in cell proliferation and migration rates among CFBE, corrected CFBE and 16HBE cells. It is worth to note that cell proliferation and migration were not assayed in wounded conditions [165], differently from other studies which nevertheless found a difference in cell migration during wound repair, as noted above. Interestingly, CFTRinh-172 induced a delay in wound repair, not only in non-CF cells (16HBE and corrected CFBE cells) but also in CFBE monolayers [165], indicating that this effect may not be related to the ion channel function of CFTR, but most probably is an unspecific effect of the inhibitor. A non-specific effect of CFTRinh-172 has also been found in other cell types of airway and non-airway origin [185]. Finally, the fact that forskolin (an activator of CFTR by raising cAMP levels) also caused delayed repair in 16HBE, CFBE and corrected CFBE cells, shed a problematic understanding of these studies. Furthermore, the data concerning CFSME and Calu-3 (obtained from different individuals) were discordant with those obtained in CF and non-CF bronchial epithelial cells, making CFSME as an inappropriate CF cell model for Calu-3 cells. Thus, since these cell lines are hardly comparable to each other due to different CFTR expression levels and to the diverse genetic background, further studies are needed to optimize culture conditions (i.e, passages) and treatment with CFTR agonists/inhibitors as well as to use wise pairs of non-CF/CF cells, as in the case of isogenic CFTR-deficient cells. Finally, the lack of conditions mimicking the in-vivo physiology (i.e., polarization and differentiation) might have affected the results.

Previous studies have indicated that *P. aeruginosa* and secreted virulence factors not only affects epithelial barrier integrity [186,187], but may also impair the ability of respiratory epithelia to repair [40,188]. More recent work has focused on the role of QS in the deleterious effects of *P. aeruginosa* exoproducts on CFTR expression and function as well as on wound repair assessed in cultures of primary non-CF and CF airway epithelial cells [41,42,189]. In particular, primary bronchial and nasal epithelial cells were evaluated as monolayers in the scratch assay [41]. The diffusible material obtained from a CF clinical strain of *P. aeruginosa* (PACF508 PsaDM) inhibited wound-repair rates in a dose-dependent fashion in non-CF monolayers. Time-lapse experiments evidence that exposure to PACF508 PsaDM resulted in a 33% decrease of migration rates, increase in cell tortuosity, and impairment in directional migration ability of cells toward the opposite side of the wounds, which is crucial for efficient epithelial repair. Interestingly, PACF508 PsaDM significantly decreased the percentage of proliferative primary non-CF airway epithelial cells at 6 h of repair. CF monolayers showed a dose-dependent inhibition of wound-repair rates in the presence of increasing concentrations of PACF508 PsaDM. The use of mutants of the QS and experiments in which LasB elastase was added convincingly showed that secreted proteases, mainly LasB elastase and secondarily LasA protease, under the control of QS are responsible for the deleterious effects on wound repair.

NE is associated with lung decline in CF [190] and uninhibited NE activity in BAL fluid (BALF) from CF infants is a strong predictor for the development of bronchiectasis [191]. To assess the role of NE in wound repair, primary bronchial epithelial cells from CF pediatric patients (monolayer cultures) were scratch wounded, and repair in the presence of NE was studied [192]. Exposure to NE (at concentrations found in CF BALF) over 72 h resulted in significantly delayed (50 nM) or inhibited (100 nM) repair of wounded CF epithelium. NE reduced both cell attachment and viability and induced apoptosis in monolayer cultures of CF and non-CF phenotypes. Moreover, NE determined a dose-dependent inhibition of proliferation that was observed for both non-CF and CF cells, whereby 50 nM NE inhibited proliferation and 100 nM abolished both attachment and proliferation of primary airway epithelial cells.

### 3.3. Wound Repair in Polarized and ALI Cultures

The expression of markers of the different cell subtypes in the airway epithelium was studied by RNA sequencing during the repair after injury of primary CF and non-CF human bronchial epithelial cells grown as ALI cultures [193]. Mechanical wounding was performed using an airbrush linked to a pressure regulator [194] (Figure 4) and RNA sequencing was performed at 24 h post-wound (pW), at wound closure (WC, ~42 h after wounding) and 2-days post-wound closure (pWC). Moreover, some cultures were also exposed to flagellin for 24 h to mimic *P. aeruginosa* infection. Gene expression of typical marker genes of the different cell subtype was monitored: TP63, cytokeratin 5 (KRT5) and KRT14 for basal cells; KRT4 and KRT13 for suprabasal cells; SCGB1A1 and SCGB3A1 for secretory cells; MUC5B and SPDEF for goblet cells; FOXJ1, FOXI1 and CFTR for ciliated cells and ionocytes. It was observed a maximal increase of basal cell markers at WC, and also of KRT4, while KRT13 showed a drop in CF cultures at the same moment. SCGB1A1 and SCGB3A1 were down-regulated at all the time points, while MUC5B was down-regulated at WC for both cultures, while it increased above the basal levels only for CF cells at pWC. FOXJ1 was also down-regulated in both cultures, whereas, interestingly FOXI1 and CFTR were under basal levels at pW and WC and at pWC returned to levels of pre-wound only in CF cultures. About RNA sequencing results, comparison of gene changes between CF and NCF cultures for the different conditions detected up- and downregulated genes in CF cells, suggesting alterations in the switch between proliferation and differentiation for CF primary cultures. Also flagellin stimulation at time 0 (non-wounded) and at WC further highlighted differences in the transcriptomic response of CF as compared to non-CF cells.

Quaresma and colleagues [195] investigated wound repair and EMT activation in CFBE cultured in a polarized way and in primary bronchial epithelial cells grown at ALI. The CFBE cells lines used in this study overexpress either wt- or F508del-CFTR, and then have the advantage of being isogenic, being the mutated CFTR the only difference among them. Both wt-CFTR expressing CFBE and non-CF fully differentiated HBE cells closed the wounds 1.5–2 times faster than the corresponding CF cells (in case of primary cells the genotype *R347P/711 +5 G > A*, a class IV mutation, was assayed). The low TEER presented by F508del-CFBE cells was recovered slowly as compared with wt-CFTR CFBE cells, confirming a delayed wound repair process in CF. Interestingly, a trend for higher proliferation (as assessed by Ki-67 staining) was found in polarized F508del-CFTR CFBE cells than in wt-CFTR-CFBE cells, while more striking results were observed for primary bronchial epithelial cells, since CF cells (with three different CFTR genotypes: *F508del*/*F508del*, *R347P/711 +5 G > A* and *M1101K*/*1609delCA*) exhibited 3-fold higher cell proliferation rates vs. control cells. The apparent discrepancy between hyper-proliferation and the wound repair defect is discussed below in the context of EMT induction.

KLF4, a transcription factor belonging to the Krüppel-like factors family, has a key role in cell-cycle regulation and epithelial differentiation [196]. Indeed, it acts as anti-proliferative gene [197]. ALI cultures obtained from CF and non-CF airway epithelial cells were investigated for Cx26 and KLF4 roles during wound repair and their reciprocal role [198]. Upon wounding of non-CF cultures, a population of basal cells in the front of repairing area showed increased expression of Ki-67, as a marker of cell proliferation, and also of Cx26. These findings were not observed at the periphery (back) of the wounded area. Similar to control cultures, in CF cells the Ki-67-labeling index increased after wounding in the front area at 12 h and reached a steady state before decreasing progressively. Interestingly, Ki-67-positive cells were readily detected in the back area of repairing CF airway epithelial cell cultures at all time points, a behavior paralleled by that of Cx26. Since GJs are growth suppressors [199], the hyper-expression of Cx26 in proliferating BCs is counterintuitive. One explanation is that Cx26 is induced in this population as a break to cell proliferation progressively favoring the formation of a tight monolayer, which may serve as a platform for later differentiation. As to KFL4, non-CF cultures showed a rapid increase in its mRNA expression and a progressive decrease over time. On the other hand, the mRNA levels for KLF4 remained unchanged during wound repair in CF cells. Finally, and quite surprisingly, CF cultures showed a faster kinetic of wound closure at 12 h and 24 h post-injury as compared to control cells, a finding that could be ascribed to the hyper-proliferative state of these cultures. Overall, these data may signify that, since Cx26 and KFL4 are both key factors in the balance between proliferation and differentiation, an altered balance of pro-and anti-proliferative signals may result in abnormal repair and remodeling of CF airway epithelial cells.

Further work was done to assess the impact of KLF4 in TEER acquisition and wound repair, using the isogenic pair wt-CFTR and F508del-CFTR-CFBE cells and their respective KLF4 knockout (KO) counterparts (via CRISPR-Cas9) [200] in polarized conditions. CF cells showed a higher proliferation rate than non-CF cells (as evaluated by Ki-67 staining and growth curve), which, however, was not influenced by KLF4 KO. Notably, KLF4 KO led to significantly less proliferation of wt-CFTR cells. As expected from other previous works, CF cells exhibited a lower TEER than their wt-CFTR corresponding controls. KLF4 KO significantly increased TEER of F508del-CFTR cells (indicating a less leaky epithelium) and delayed wound closure, whilst had the opposite effect on wt-CFTR CFBE cells, i.e., decreased TEER. The wound closure in non-CF cells was not altered by KLF4 KO, showing a differential effect of KFL4 KO in the two cell lines. Although a different actin cytoskeleton organization was observed by comparing wt- to F508del-cells, no alteration in lamellipodia formation was observed in the presence of KLF4 KO. Sousa and colleagues [200] showed that KLF4 KO decreases wound closure even further in F508del-CFTR cells (although not significantly), suggesting that KLF4 promotes wound repair. On the other hand, the wound closure in non-CF cells was not altered by KLF4 KO. The authors speculated that since CF cells start from a more mesenchymal state [17,18], this may influence the outcome of KLF4 KO. It has been shown a high expression of KLF4 in the same CF cell line as compared with wt cells [201]. However, KLF4 down-regulation promoted expression of wt-CFTR but not of F508del–CFTR, indicating a specific impact of KLF4 on normal CFTR. Moreover, in primary CF airway epithelial cells grown at ALI, the mRNA levels for KLF4 remained unchanged during wound repair [198]. Overall, these data indicate that if KLF4 have an impact on CF epithelial wound repair this is subtle, and the reason could be that there might be some compensatory mechanism between KFL4 and other KLFs (KLF5 and KLF2), as well as some degree of redundancy [201].

### 3.4. Cellular and Molecular Events in CF Wound Repair

The delayed repair of mechanically-injured CF cells (CuFi) was shown to be dependent on the decreased levels of EGF secretion and/or EGFR (erbB1 and erbB2) activation (evaluated as phosphoprotein) [162]. It was also established that K^+^ currents were lower in CuFi as compared with NuLi cells, due to decreased electrophysiological activities of K^+^ channels (KvLQT1, K_ATP_, and KCa3.1). Interestingly, it was shown that EGF-stimulated wound repair of NuLi and CuFi monolayers depends on K^+^ channel activity. Since EGF application to NuLi cells increased KvLQT1-mediated K^+^ currents but not in CuFi cells, these results indicate that K^+^ channels controlled EGF-dependent wound repair and that this axis is deficient in CF cells. In the process of cell migration with lamellipodia formation in the front of cells and cell retraction in the rear, K^+^ channels might be involved with anion channels (e.g., CFTR) and cationic channels (e.g., ENaC), to cell volume changes at the leading (swelling) or rear (shrinking) edges of migrating cells [202]. Indeed, activation of a Cl^−^ conductance may be important for controlling cell volume at the trailing edge of migrating cells as a mechanism that contribute to the extension of the lamellipodium [203]. It has been shown that inhibition of certain K^+^ channel subtypes slows the rate of cell migration [204]. K^+^ channels participate in regulation of cell volume by providing a pathway for K^+^ exit at the rear of the cell, creating a driving force for water efflux [203,204]. Indeed, increasing volume and membrane tension at the front of the cell eventually trigger a rise of intracellular Ca^2+^ concentration ([Ca^2+^]i) via activation of Ca^2+^-permeable, stretch-activated cation channels. The rise of [Ca^2+^]i induces the retraction of the rear part of a migrating cell, which is paralleled by massive K^+^ efflux and local cell shrinkage at the rear [203]. The importance of K^+^ channels in migration presumably necessitates Cl^−^ channel activation as a means of sustaining K^+^ transport and to provide additional solute efflux to enhance the osmotic driving force. A study in favor of this hypothesis showed that endogenous electric currents at sites of tracheal epithelial injury may direct cell migration, an observation that was substantiated by the reduction of wound currents inhibiting CFTR with CFTR(Inh)-172 [205]. An alternative hypothesis about the role of CFTR was proposed by Schiller and colleagues [163], who built on the known HCO_3_^-^ transport activity of CFTR [206,207]. CFTR-mediated HCO_3_^-^ efflux at the rear of the cell could determine local alkalinization, reduce adhesion, and enhance migration. Moreover, intracellular acidification may sustain activity of the Na^+^/H^+^ exchanger (NHE1), a well-known regulator of cell spreading and lamellipodia formation [208,209]. Overall, these data support an important role of ion transporters in airway epithelial wound closure, especially CFTR.

Further studies showed the role of the pro-inflammatory cytokine TNF-α in airway epithelial wound repair [138]. Chronic treatment with TNF-α (24–48 h) accelerated both NuLi and CuFi wound closure, whereas an acute treatment (6 h) had only slight enhancing effects. The stimulation of the wound-repair rate was likely due to an increase in bronchial cell migration, despite reduced cell growth induced by TNF-α. Interestingly, it was observed that basal and TNF-α stimulation of the migration rate was lower in CuFi than in NuLi. To investigate the role of different types of metalloproteinases (MMPs and ADAMs) in wound repair enhancement by TNF-α, a MMP broad-spectrum inhibitor (GM6001) was used and shown to reduce Nuli and CuFi repair rates. MMPs were likely involved in EGF shedding from the plasma membrane and autocrine activation of EGFR. The involvement of K^+^ channels was also determined, as K^+^ channel inhibition abolished the wound repair stimulation evoked by TNF-α. All in all, based on these and previous results [138,162], it is conceivable to propose the following model: TNF-α induces secondary MMP-9 and EGF release, followed by EGFR activation, thus stimulating NuLi and CuFi cell migration and wound repair, possibly through the stimulation of K^+^ channels.

Another study [166] focused on the role of sphingolipids gangliosides in CF-silenced cells [163] and the delayed repair of an epithelial wound. The interaction of gangliosides with integrin regulates cell adhesion and migration [210]. CFTR-silenced Calu-3 cells displayed significantly lower levels of the ganglioside GM1. Other genetic and pharmacologic approaches confirmed that CFTR function is required to maintain normal levels of GM1 in this cell line. Activation of β_1_-integrin and downstream focal adhesion kinase (FAK) and p130/Crk-associated substrate (CAS) phosphorylation were found to be suppressed in CF-silenced cells. Importantly, GM1 addition to CF-silenced cells restored activation of β_1_-integrin, FAK and CAS phosphorylation, as well as led to a significantly increased rate of migration. Thus, it may be inferred that a reduction in GM1 in CF cells (through a yet unexplained mechanism) contributes to lower cell migration and wound closure rate via inhibition of β_1_-integrin activation and signaling. More recently, it has been shown that an active β_1_ integrin is trapped in the luminal pole of bronchial and nasal epithelial cells of CF individuals in vivo and in vitro, leading to the downregulation of the expression of acid ceramidase in human CF airway epithelial cells and thereby mediating a further accumulation of ceramide and a concomitant depletion of sphingosine [211]. Since sphingosine plays a key role in the innate and immediate defense of the upper respiratory tract, these findings could explain the high susceptibility of CF patients to opportunistic bacterial infections, in particular *P. aeruginosa*. The Chanson’s group has recently demonstrated that the CF-dependent ectopic expression of β1 integrin at the apical surface of CF cells is a consequence of Vav3 (a guanine nucleotide exchange factor) overexpression [212]. This phenotype was involved in a disorganized pattern of actin cytoskeleton, higher fibronectin expression at the same location, and remodeling at the surface of CFTR-silenced Calu-3 cells associated with enhanced binding of *P. aeruginosa*. However, silencing Vav3 did not affect Calu-3 cell migration as observed in a wound repair assay. Though β1 integrin expression is increased and apically exposed during normal airway epithelium reparation, a process associated with higher fibronectin production [69], its role in wound repair as apical protein has not been deciphered yet. Similarly, it is not clear at the moment if Vav3 hyper-expression in CF epithelial cells is only involved in *P. aeruginosa* higher binding to and colonization of airway epithelial cells or is also participating in deregulated wound repair processes.

**Figure 5:**
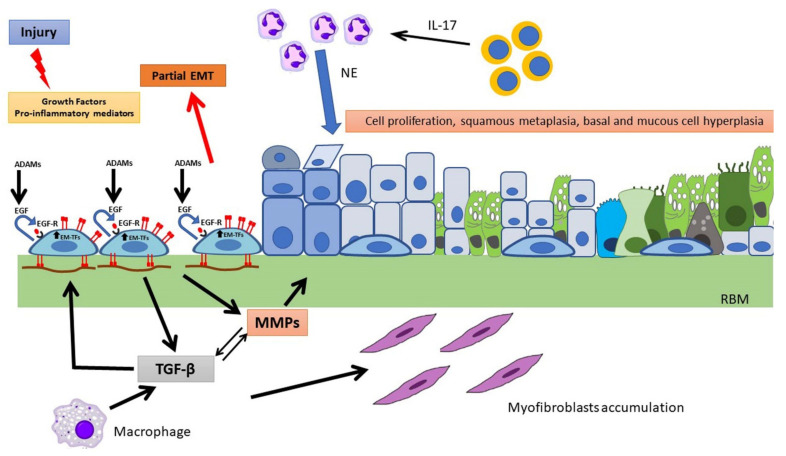
Wound repair and regeneration in a CF airway epithelium. The pro-inflammatory milieu of the CF airways and the partial EMT state of CF airway epithelial cells (as represented by the detachment of a single cell from the epithelial sheet)) stimulate processes involved in regeneration and repair that are exaggerated in regard to a non-CF epithelium (as one can observe by the arrow thickness in comparison to Figure 2). However, the wound repair does not occur properly, eventually leading to persistence of cell proliferation, squamous metaplasia, and basal and mucus cell hyperplasia. The thickening of reticular basement membrane (RBM) is likely due to elevated TGF-β levels and enhanced myofibroblast differentiation and accumulation. Neutrophils infiltrate the CF airways following IL-17 secretion by T cells and produce mediators (e.g., NE) that retard the wound repair.

EMT involves transcriptional reprogramming of epithelial cells into a mesenchymal phenotype with enhanced migratory capacities, which has been studied in relation with cancer invasiveness and inflammatory diseases [213]. Chronic obstructive pulmonary disease (COPD) and idiopathic pulmonary fibrosis (IPF) are chronic lung diseases which also present EMT associated with abnormal repair and tissue remodeling/fibrosis [214,215,216]. A transcriptomic analysis revealed that altered epithelial differentiation and EMT are active in CF airways [217]. A recent paper from Amaral’s research group [195] clearly shows that native lung tissue from CF patients have increased transcript levels of epithelial markers, such as those of TJs (occludin and ZO-1), GJs (Cx43, Cx26), and cytokeratin 18 (CK18) as well as have increased mesenchymal marker expression (vimentin) as compared with non-CF tissues. However, other epithelial markers (claudin, E-cadherin, β-catenin, Cx31 and desmoplakin) were not changed, as also other mesenchymal markers (N-cadherin and fibronectin). On the other hand, immunofluorescence analysis revealed that CF epithelia presented a disorganized structure with multiple layers resembling a condition of squamous metaplasia and a weak expression of epithelial markers with an increase in mesenchymal markers. Overall, these results show that despite increased transcription of epithelial markers, there is a disorganized patter of their expression (although not of all of them) that, along with increased mesenchymal marker expression, indicate a partial EMT activation in vivo. Similarly to CF tissues, fully differentiated primary CF bronchial epithelial cells show increased levels of mesenchymal markers (N-cadherin and vimentin), although not accompanied by decreased levels of epithelial markers (CK18, ZO-1 and E-cadherin), and correspondingly have a decreased TEER, suggesting impaired cell-cell contacts. Polarized F508del-CFTR cells presented some features observed in bronchial tissues, i.e., they were multilayered, the expression of E-cadherin was less confined to AJs, deposited more collagen I to the basal extracellular matrix and expressed increased levels of diffusely localized N-cadherin vs. wt-CFTR cells. Interestingly, the triple combination of two correctors (VX-445, VX-661) and a potentiator (VX-770) efficiently down-regulated the expression of vimentin and N-cadherin in F508del-CFBE cells. To test the hypothesis that CFTR plays an important role in protecting against EMT induction, F508del-CFBE and wt-CFBE cells were treated with TGFβ-1, a known inducer of EMT in cultured airway epithelial cells [218]. While a similar decrease in CK18 and increase in N-cadherin expression was observed in the two cell lines, F508del-CFTR CFBE cells displayed a higher decrease in E-cadherin and increase in vimentin. Interestingly, F508del-CFTR rescue by the triple combo partially blocked the TGF-β1-mediated EMT induction. Moreover, treatment of wt-CFTR expressing cells with the triple drug combination completely blocked TGF-β1-induced EMT. Altogether, these data provide evidence for resistance to EMT conferred by the wt-CFTR and that dysfunctional CFTR is associated with EMT induction. Transcript analysis and immunofluorescence showed upregulation of TWIST1, Snail+Snug and ZEB1 EMT-TFs in CF vs. non-CF lung tissues. TWIST1 was found to be significantly increased in F508del-CFTR CFBE cells. Treatment of F508del-CFTR with VX-445/VX-661/VX-770 determined a decrease in TWIST1 but not in Snail+Snug protein expression. TWIST knock-down by shRNA in F508del-CFTR CFBE cells was enough to determine inhibition of vimentin expression both in presence and absence of TGFβ-1. As CF cells display increased proliferation and altered wound repair, as discussed in the previous Section, these features have to be reconciled with all the other observations, i.e., mislocalization of cell junction proteins, disruption of epithelial architecture, aberrant expression of mesenchymal markers and EMT-TF upregulation. The alteration in wound repair, although consistent with previous reports on this topic, was quite an unexpected finding, since mesenchymal cells show a migratory phenotype not presented by epithelial cells, and hyper-proliferative and migratory/invasive behavior are characteristics of EMT. Indeed, the phenotype characteristics are appropriate for a partial EMT activation in CF, also occurring in cancer, fibrosis and other chronic lung diseases [216,219,220]. In the partial CF EMT, cells still migrate mostly as monolayers as observed by live cell imaging of wound closure, like epithelial cells do. Thus, it is likely that the CF TJ defects could account for the observed CF delay in wound closure, given the importance of cell-to-cell contacts in cell migration. In parallel, mutant CFTR can cause increased proliferation while being detrimental to tissue regeneration, as reported [15,163]. Finally, increased migration does not necessary occur in EMT [221]. Thus, the partial CF EMT behavior, characterized by TJ dysfunction and low migratory phenotype, may explain the defect in wound repair. It should be considered that besides the altered CFTR-TWIST1 axis, that incites a first hit, other pathological conditions found in CF airways may advance CF cells towards the partial EMT condition, such as chronic inflammation, i.e., increased TGF-β1 [121], epithelial remodeling, pathogen invasion [15], hypoxia and aberrant myofibroblast persistence [129] (Figure 5).

Sousa and colleagues [200] investigated in polarized models of bronchial epithelial cells (wt- vs. F508del-CFTR CFBE cells) the effect of KLF4 KO on proliferation and wound repair (as discussed above) as well as the modulation of differentiation markers and EMT induction. KLF4 KO decreased the levels of E-Cadherin and CK18, whilst increasing the levels of vimentin and N-cadherin in both cell lines. However, the effect on fibronectin and ZO-1 was different. KLF4 KO increased the expression levels of fibronectin in wt-CFTR cells, whereas it led to a decrease in fibronectin in F508del-CFTR cells. In regard to ZO-1, KLF4 KO decreased its expression in F508del-CFTR CFBE cells, having no major impact on wt-CFTR CFBE cells. Besides the finding that TWIST1 expression was upregulated in CF vs. non-CF cells, the effect of KLF4 KO on the expression of EMT-TFs differed between the two cell lines. Indeed, while KLF4 KO produced a marked decrease of TWIST1 in F508del-CFTR CFBE cells, it had no major impact on its levels in wt-CFTR CFBE cells. As to the signaling of TGFβ-1, KLF4 KO also promoted the expression of TGF receptors I and II only in wt-CFTR CFBE cells, with an increasing trend for F508del-CFTR CFBE cells. Taken together, it is conceivable to assert that KLF4 has different effects depending on the CFTR state of the cells. Overall, these data are compatible with the observation that CF cells are already more mesenchymal than wild type-CFTR cells, as previously observed [195]. Since KLF4 has been shown to prevent EMT [47], the effect of its KO is in accordance with what was observed for the modulation of E-cadherin, N-cadherin, and vimentin. However, due to the other effects of KLF4 KO, such as the upregulation of fibronectin and the downregulation of SMAD7 and TWIST1, the emerging EMT behavior that KLF4 KO causes in F508del-CFTR cells, is possibly compensated through a feedback mechanism to retain epithelial function. Further studies are warranted to elucidate the role of KLF4 in CF relevant pathophysiological aspects, such as disrupted differentiation and regeneration as well as in the EMT activation.

## 4. Modulation of Wound Repair in CF

Since wound repair is part of the pathophysiology process occurring in CF airways, its modulation would be paramount for therapeutic interventions on epithelium reconstitution. Some studies have reported an augmentation in CF wound repair and they will be hereafter described.

Inhibition of wound repair by NE in primary CF monolayer cultures could be rescued by exposure to alpha-1 antitrypsin (α1AT), the major host antiprotease, when the inhibitor was added even after 12 h of exposure to 50 nM NE, and restoring wound closure to control levels by 30 h [192]. α1AT also reversed the loss of adhesion due to 100 nM NE, resulting in reattachment and significantly increased wound closure by 72 h.

A breakthrough in the management and therapy of people with CF has been the approval and introduction in the clinic of small-molecule drugs that target the root of CF disease [222]. Correctors are small molecules that allow the mutant CFTR protein to transit from endoplasmic reticulum to the plasma membrane (class II), while potentiators increase channel activity of a mutant CFTR already correctly positioned in the apical membrane (class III, IV and V). Besides their positive effects on sweat chloride and respiratory function, CFTR modulators have been shown to influence epithelial immune responses [223]. VRT-325, a corrector of mutated F508del-CFTR [224], was able to accelerate wound closure in CFBE-F508del monolayers to a rate similar of that presented by CFBE expressing wild type-CFTR [164]. Interestingly, wound-repair rates in primary bronchial cell monolayers were also increased by 1.8 fold in average by VRT-325. Orkambi^®^ is combination of a corrector (lumacaftor [VX-809]) and a potentiator (ivacaftor [VX-770]) that has proved higher efficacy in CF patients homozygous for *F508del* [225] than lumacaftor alone [226]. Airway epithelial repair was studied in CF subjects homozygous for *F508del* and in those heterozygous for *F508del* and another class II mutation (*N1303K* or *I507del*) [184]. In all monolayers with wounds generated by the scratch method, Orkambi^®^ increased wound closure as compared to untreated controls and lumacaftor-treated cells. The repair improvement by Orkambi^®^ was abrogated by the CFTR inhibitor GlyH101, confirming that this effect is dependent on CFTR function, although off target effects cannot be ruled out [227]. While the presence of *P. aeruginosa* exoproducts impaired wound repair rates of CF airway epithelial cell monolayers, confirming previous results [41], Orkambi^®^ heightened the repair rates, although at levels lower than in the absence of infection. To better mimic the in vivo situation, highly differentiated cultures of primary airway epithelial cells were developed at ALI and injured by a vacuum-dependent method. Orkambi^®^ treatment accelerated the repair under these conditions in the absence and presence of *P. aeruginosa* exoproducts.

Human mesenchymal stem cells are a source for regenerative medicine approaches aimed to treat many pathological conditions arising from either congenital or acquired conditions, including lung diseases [228,229,230]. Their regenerative capacities towards repair of lung injuries and reduction of inflammation is likely to be due to cell-to-cell contact, mitochondrial transfer, and release of soluble mediators or extracellular vesicles [231]. We employed a novel strategy to treat the basic defect in CF by using MSCs obtained from an ethical source, i.e., the amniotic membrane at the moment of parturition [232]. Human amniotic MSCs (hAMSCs) in co-culture with CFBE41o- cells (*F508del* homozygous) rescued CFTR activity, TJ and actin organization, as well as reduced hyperactivity of ENaC [233]. More recently, we have demonstrated that hAMSCs form GJs with CFBE cells and that siRNA-mediated downregulation of Cx43 abolishes the rescue of CFTR activity by hAMSCs [234]. In order to gain more insight in the application of hAMSCs in an in vivo setting, we have preliminarily studied the effect of hAMSCs in the wound closure of a CFBE41o- monolayer injured by a pipette scratch. CFBE41o- were slower in the wound closure than control wt-CFTR cells (i.e., 16HBE14o-), whereas the wound closed faster either in the co-culture system or when hAMSCs were added to the injured monolayers (unpublished results). These results encourage us to think that if MSCs could be applied to injured CF lungs via a topical application they would favor the repair of an abnormally inflamed airway epithelium.

## 5. Conclusions

It is unquestionable that wound repair is affected in CF, as shown by different cellular models. Continuous injury by bacteria and their exoproducts, as well as chronic exposure to inflammatory cytokines may render CF cells less amenable to migration, thereby impeding or decelerating wound closure. A role in this alteration would be a deranged EGF-EGFR-K^+^ channel axis, worsened by the hyper-inflammatory milieu. Another role could be played by the interplay between the EGFR and the transmembrane proteases ADAMs, sheddases with multiple targets (AREG and IL-6 receptor) and whose activity can be enhanced by injury and can play a pathological role in tissue remodeling and inflammation [142]. Although not verified in the wound closure, it was shown that CFBE41o- cells grown at ALI showed enhanced ADAM17-mediated shedding of AREG and increases phosphorylation of EGFR [146].

Other issues have to further studied. It is undisputable from data emerging from the use of CFTR-deficient cells, CFTR inhibitors, CFTR gene silencing, and CFTR modulators that CFTR plays a key role in the regeneration and repair of the airway epithelium. Results within some studies point to its positive effect on these processes as dependent on its functional activity as an ion channel, while it is not known whether CFTR may regulate repair and regeneration of the airway epithelium via its interaction with other proteins and hence influence other cellular functions. Another issue that it would be worth to explore is a link between CFTR defect, cytoskeleton remodeling and wound repair in CF, shedding light on the establishment of cell polarity during the migration process and directionality (front vs. back). This assumption is also linked to indirect effects of CFTR as silencing CFTR reduces GM1 and cholesterol expression leading to alteration of β_1_ integrin-mediated cell adhesion, which presumably affects the ability of cells to achieve sufficient traction to support migration. Another question would be in regarding to the inflammatory mediators: do they play a role at the onset of CF airway disease or are they an epiphenomenon? Are there other essential inflammatory mediators and immune molecules that we are missing from the whole picture? It is also essential in this context to understand the role of bacterial infections: is it better to intervene on bacteria when they are planktonic to prevent biofilm-forming bacteria? And related to MSCs: would it better to deliver MSCs to CF lungs in their native state or already differentiated into epithelial cells? Answers to these questions would essential to better direct therapies (CFTR modulators, anti-inflammatory and immune modulatory drugs, QS inhibitors, stem cells) aimed at resolving the wound repair defect associated to CF at the level of the airway epithelium.

## Figures and Tables

**Table 1:** Classification of CFTR mutations and phenotype severity.

Mutation Class	Class I		Class II	Class III	Class IV	Class V	Class VI
	IA	IB					
CFTR defect	No mRNA	No protein	No traffic	Impaired gating	Decreased conductance	Less protein	Less stable
Mutation example	Dele2,3(21 kb), 1717-1G→A	G542X, W1282X, 1609delCA	Phe508del, N1303K, M1101K	G551D, S549R, G1349D	R117H, R334W, A455E	3272-26A→G, 3849+10 kg C→T	c. 120del123, rPhe580del
Phenotype severity	More-severe disease				Less-severe disease		

Adapted from refs. [3,4]. For each mutation, the legacy name is reported according to the Cystic Fibrosis Mutation Database (http://www.genet.sickkids.on.ca/ (accessed on 4 March 2021).

**Table 2:** In vitro models used to study wound repair and its modulation in CF.

Cellular Models	Type of Culture	Type of Wound	Effects on Wound Closure	Effects on Proliferation/Migration	Modulation	Reference
Immortalized cell lines: normal (NuLi) and CF (CuFi-1, *F508del* homozygous) bronchial cells	Submerged on plastic	Mechanical injury of monolayers (pipette tip scratch assay). Wound closure was evaluated by light microscopy.	CuFi-1 monolayers showed a slower wound repair up to 33% as compared with NuLi in the absence or presence of exogenous EGF.	CuFi-1 cells exhibited slower migration (by 25%) than NuLi cells. CuFi-1 cells showed a proliferation rate similar to NuLi cells.	Inhibition of K^+^ channels decreased EGF-stimulated wound repair in both cell lines. CFTRinh-172 did not affect significantly wound closure in both cell phenotypes.	Trinh et al., 2008 [162]
Immortalized cell lines: Calu-3 (normal human lung adenocarcinoma cell line); UNCCF1T (CF human bronchial epithelial cells; *F508del* homozygous). Primary cell lines: NHBE (normal human bronchial epithelial cells).	Submerged on plastic	Circular lesion produced by lethal electroporation. Wound closure was measured by continuous impedance sensing (CIS) combined with phase-contrast imaging.	UNCCF1T showed delayed wound closure as compared with NHBE cells	UNCCF1T showed slower cell migration (by 1.7-fold) as compared with NHBE cells	CFTRinh-172 delayed wound closure of both Calu-3 and NHBE cells. A CFTR-specific shRNA (shCFTR) delayed wound closure in Calu-3 cells.	Schiller et al., 2010 [163]
Immortalized cell lines: NuLi and CuFi-1 cells. Primary cell lines: non-CF and CF human airway epithelial cells (from nasal polyps)	Submerged on plastic	Mechanical injury of monolayers (pipette tip scratch assay). Wound closure was evaluated by light microscopy.	CuFi-1 monolayers showed a slower wound-repair rate as compared with NuLi in the absence or presence of exogenous EGF. CF primary airway monolayers showed a reduced wound-repair rate as compared with non-CF monolayers.	CuFi-1 cells showed a proliferation rate similar to NuLi cells.	TNF-α chronic exposure (24–48 h) enhanced CuFi-1 and NuLi wound-repair rate in the absence and presence of exogenous EGF. TNF-α increased cell migration in wounded NuLi and CuFi-1 monolayers, despite inhibition of cell growth. TNF-α stimulated wound repair in non-CF and CF primary monolayers.	Maille et al., 2011 [138]
Immortalized cell lines: NuLi and CuFi-1 cells; IB3 (*F508del*/*W1282X*) and S9 (wt-CFTR genetically repaired IB3 cells); CFBE41o- transduced with wt-CFTR (CFBE-wt) or F508del-CFTR (CFBE-F508del). Primary cell lines: non-CF and CF bronchial and nasal epithelial cells.	Submerged on plastic	Mechanical injury of monolayers (pipette tip scratch assay). Wound closure was evaluated by light microscopy	CuFi-1 monolayers showed a slower wound-repair rate as compared with NuLi. The wound closure rate of CF human bronchial cell monolayers was 63% slower than that of non-CF bronchial monolayers. The wound closure in CF human nasal monolayers was delayed when compared with non-CF (53% slower wound-repair rate). S9 and CFBE-wt showed a higher wound-repair rate than IB3 and CFBE-F508del, resepctively.	CuFi-1 cells exhibited slower migration (by 25%) than NuLi cells. CuFi-1 cells showed a proliferation rate similar to NuLi cells.	CFTR silencing by siRNA and the CFTR inhibitor GlyH101 elicited a significant decrease in wound repair in non-CF primary nasal epithelial cell monolayers. CFTR inhibition by GlyH101 reduced significanlty both cell migration and proliferation in non-CF primary nasal epithelial cell monolayers. The CFTR corrector VRT-325 enhanced the wound repair rate of CFBE-F508del and CF primary bronchial epithelial cell monolayers.	Trinh et al., 2012 [164]
Immortalized cell lines: 16HBE14o-(wt CFTR) (normal human bronchial epithelial cells; CFBE41o-(*F508del* homozygous), and its corresponding plasmid-corrected CFBE41o-pCep4, overexpressing wtCFTR) cells; Calu-3 and CFSMEo- (CF submucosal gland epithelial cells, *F508del*/*2QX*) cells	Submerged on plastic	Mechanical injury of monolayers (pipette tip scratch assay). Wound closure was evaluated by light microscopy	CFBE cells showed a slower wond closure compared to corrected CFBE cells but not in resepct to 16HBE cells. CFSME showed a significant delay in wound repair time compared to Calu-3 cells	CFBE, corrected CFBE, and 16HBE showed the same proliferation and migration rates in non-wounded conditions.	CFTRinh-172 and forskolin induced a delay in wound repair in 16HBE, CFBE and corrected CFBE cells.	Hussain et al., 2014 [165]
Immortalized cell lines: CFTR-silenced (shRNA) and control Calu-3 cells.	Submerged on plastic	Circular lesion produced by lethal electroporation. Wound closure was measured by continuous impedance sensing (CIS) combined with phase-contrast imaging.	CFTR-silenced cells showed a reduced rate of electrode coverage as compared with control cells.	Cell migration was slower in CFTR-silenced cells than in control cells.	Ganglioside GM1 partially restored the wound-repair defect in CFTR-silenced cells.	Itokazu et al., 2014 [166]
Primary cell lines: non-CF and CF airway epithelial cells (MucilAir™ and MucilAir™-CF)	ALI cultures	Circular wounds obtained by an airbrush linked to a pressure regulator. Wound closure was evaluated by light microscopy.	CF cultures showed a higher wound repair rate than non-CF cultures at early time pints (12 and 24 h).	In non-CF cultures, the Ki-67-labeling index reached its maximum at 48 h post-wounding and was higher in the front area as compared with the front area. In CF cultures, the maximum of the Ki-67-labeling index was reached at 36 h, with no significant difference between front and back areas.	In non-CF cultures, CX26 mRNA expression paralleled the behavior of Ki-67 labeling index. Also KLF4 showed a transient increase during the early timepoints after injury. In CF cutlures, the increase in Cx26 mRNA expression peaked at 60 h post-wounding, with no significant difference between front and back areas. KLF4 mRNA levels remained unchanged during wound repair.	Crespin et al., 2014 [198]
Primary cell lines: non-CF and CF nasal and bronchial epithelial cells.	Submerged on plastic and ALI cultures	Mechanical injury of monolayers (pipette tip scratch assay). Wound closure was evaluated by light microscopy.	*P. aeruginosa* exoproducts inhibited wound-repair rates in non-CF cells, affected cell trajectories and impaired their directional migration ability toward the opposite side of the wounds. A dose-dependent inhibition of wound-repair rates in CF cells was observed in the presence of increasing concentrations of *P. aeruginosa* exoproducts.	*P. aeruginosa* exoproducts decreased the percentage of proliferative primary non-CF cells at 6 h of repair.	Quorum sensing inihibitor HDMF can reduce the exoproducts-induced wound-repair defect of non-CF and CF monolayers and as well as of highly differentiated cell cultures.	Ruffin et al., 2106 [41]
Primary cell lines: non-CF and CF bronchial epithelial cells	Submerged on plastic	Mechanical injury of monolayers (WoundMaker device). Wound closure was evaluated by IncuCyte live-cell imaging system.	NE exposure determined a dose-dependent delay/inhibition of wound repair at 30–72 h.	NE exposure caused increased cell detachment of viable cells, reduction in cell viability, apoptosis, and reduction in cell proliferation.	Wound closure by CF cells initially exposed to 100 nM NE and then treated with 1 mM α1AT was significantly increased over 100 nM NE alone.	Garratt et al., 2016 [192]
Primary cell lines: non-CF and CF bronchial and nasal epithelial cells. CF cells were obtained from 4 homozygous *F508del* patients and 4 patients carrying *F508del* and another class II mutation (*N1303 K* or *I507del*).	Submerged (monolayers) and ALI cultures	Monolayers: mechanical injury (pipette tip scratch assay). Wound closure was evaluated by light microscopy. ALI cultures: mechanical wound by a glass Pasteur pipette connected to the vacuum. Wound closure was evaluated by time-lapse microscopy.	CF ALI cultures showed a delay in wound repair as compared with non-CF cultures.	Proliferation and migration were not assessed.	In all patients, the improvement in the wounding repair rate over a 6h-period of monolayers was higher after CFTR rescue with Orkambi^®^ (VX-809 + VX-770), compared to VX-809 alone. Orkambi^®^ treatment sighltly improved the repair rates of CF monolayers in the presence of *P. aeruginosa* exoproducts. Similar results were obtained with CF differentiated cultures.	Adam et al., 2018 [184]
Primary cell lines: non-CF and CF bronchial epithelial cells.	ALI cultures	Circular wounds obtained by an airbrush linked to a pressure regulator.	Significant differences in gene expression of different cell types between CF and non-CF cultures at post-wound, wound closure and post-wound closure were not observed. Comparison of gene expression by RNA sequencing determined that CF cultures had up- and down-regulated genes as compard with non-CF cultures at all the conditions (non-wounded, post-wounding, wound closure, and post-wound closure).	Both cultures showed high proliferation during wound as assessed by the expression of MKI67 gene.	*P. aeruginosa* flagellin determined up- and down-regulation of genes when CF and non-CF cultures were compared pre-wounding and at wound closure.	Zoso et al., 2019 [193]
Immortalized cell lines: CFBE41o- cells stably overexpressing wt- or F508del-CFTR. Primary cell lines: non-CF and CF bronchial cells. epithelial cells	Polarized on filter (CFBE cell lines) or ALI cultures (primary bronchial cells).	Mechanical injury of monolayers (pipette tip scratch assay). Wound closure was evaluated by light microscopy.	wt-CFTR CFBE and fully differentiated bronchial epithelial cells closed the wounds 1.5–2 times faster than corresponding CF cells.	Primary CF bronchial cells (three different CFTR genotypes) exhibiting 3-fold higher cell proliferation rates vs control cells.	The triple drug combo VX-445/VX-661/VX-770 restored the epithelial phenotype in F508del-CFTR CFBE reducing mesenchymal cell markers, but no effect on wound repair rate was assessed.	Quaresma et al., 2020 [195]
Immortalized cell lines: CFBE41o- cells stably overexpressing wt- or F508del-CFTR.	Polarized on filter.	Mechanical injury of monolayers (pipette tip scratch assay). Wound closure was evaluated by light microscopy.	wt-CFTR CFBE closed the wounds faster than corresponding CF cells, although significance was not assessed.	CF cells showed higher proliferation than non-CF cells	KLF4 KO had no major impact on cell proliferation in the CF context. KLF4 KO significantly decreased TEER of wt-CFTR cells whilst increasing TEER of F508del-CFTR cells. KLF4 KO in F508del-CFTR cells determined a delay in wound closure, while having no effect on wt cells.	Sousa et al., 2020 [200]

α1AT: alpha-1 antitrypsin; HDMF: 4-hydroxy-2,5-dimethyl-3(2H)-Furanone; NE: neutrohil elastase.

## Data Availability

Data sharing not applicable.
